# Effect of chronic intermittent hypoxia (CIH) on neuromuscular junctions and mitochondria in slow- and fast-twitch skeletal muscles of mice—the role of iNOS

**DOI:** 10.1186/s13395-022-00288-7

**Published:** 2022-02-12

**Authors:** L. I. Bannow, G. A. Bonaterra, M. Bertoune, S. Maus, R. Schulz, N. Weissmann, S. Kraut, R. Kinscherf, W. Hildebrandt

**Affiliations:** 1 Department of Medical Cell Biology, Institute of Anatomy and Cell Biology, Philipps-University Marburg, Robert-Koch-Straße 8, 35032 Marburg, Germany; 2 Department of Pulmonary Medicine, Helios Dr. Horst Schmidt Clinic, Wiesbaden, Germany; 3 Justus-Liebig University of Giessen (JLUG), Excellence Cluster Cardiopulmonary Institute (CPI), Universities of Giessen and Marburg Lung Center (UGMLC), member of the German Center for Lung Research (DZL), Giessen, Germany

**Keywords:** Denervation, Muscle atrophy, Mitochondria, Fiber type, Oxidative stress, Neuromuscular junction, iNOS

## Abstract

**Background:**

Obstructive sleep apnea (OSA) imposes vascular and metabolic risks through chronic intermittent hypoxia (CIH) and impairs skeletal muscle performance. As studies addressing limb muscles are rare, the reasons for the lower exercise capacity are unknown. We hypothesize that CIH-related morphological alterations in neuromuscular junctions (NMJ) and mitochondrial integrity might be the cause of functional disorders in skeletal muscles.

**Methods:**

Mice were kept under 6 weeks of CIH (alternating 7% and 21% O_2_ fractions every 30 s, 8 h/day, 5 days/week) compared to normoxia (NOX). Analyses included neuromuscular junctions (NMJ) postsynaptic morphology and integrity, fiber cross-sectional area (CSA) and composition (ATPase), mitochondrial ultrastructure (transmission-electron-microscopy), and relevant transcripts (RT-qPCR). Besides wildtype (WT), we included inducible nitric oxide synthase knockout mice (iNOS^−/−^) to evaluate whether iNOS is protective or risk-mediating.

**Results:**

In WT soleus muscle, CIH vs. NOX reduced NMJ size (− 37.0%, *p* < 0.001) and length (− 25.0%, *p* < 0.05) together with fiber CSA of type IIa fibers (− 14%, *p* < 0.05) and increased centronucleated fiber fraction (*p* < 0.001). Moreover, CIH vs. NOX increased the fraction of damaged mitochondria (1.8-fold, *p* < 0.001). Compared to WT, iNOS^−/−^ similarly decreased NMJ area and length with NOX (− 55%, *p* < 0.001 and − 33%, *p* < 0.05, respectively) or with CIH (− 37%, *p* < 0.05 and − 29%, *p* < 0.05), however, prompted no fiber atrophy. Moreover, increased fractions of damaged (2.1-fold, *p* < 0.001) or swollen (> 6-fold, *p* < 0.001) mitochondria were observed with iNOS^−/−^ vs. WT under NOX and similarly under CIH. Both, CIH- and iNOS^−/−^ massively upregulated suppressor-of-cytokine-signaling-3 (SOCS3) > 10-fold without changes in IL6 mRNA expression. Furthermore, inflammatory markers like CD68 (macrophages) and IL1β were significantly lower in CIH vs. NOX. None of these morphological alterations with CIH- or iNOS^−/−^ were detected in the gastrocnemius muscle. Notably, iNOS expression was undetectable in WT muscle, unlike the liver, where it was massively decreased with CIH.

**Conclusion:**

CIH leads to NMJ and mitochondrial damage associated with fiber atrophy/centronucleation selectively in slow-twitch muscle of WT. This effect is largely mimicked by iNOS^−/−^ at NOX (except for atrophy). Both conditions involve massive SOCS3 upregulation likely through denervation without Il6 upregulation but accompanied by a decrease of macrophage density especially next to denervated endplates. In the absence of muscular iNOS expression in WT, this damage may arise from extramuscular, e.g., motoneuronal iNOS deficiency (through CIH or knockout) awaiting functional evaluation.

**Supplementary Information:**

The online version contains supplementary material available at 10.1186/s13395-022-00288-7.

## Introduction

**Fig. 1:**
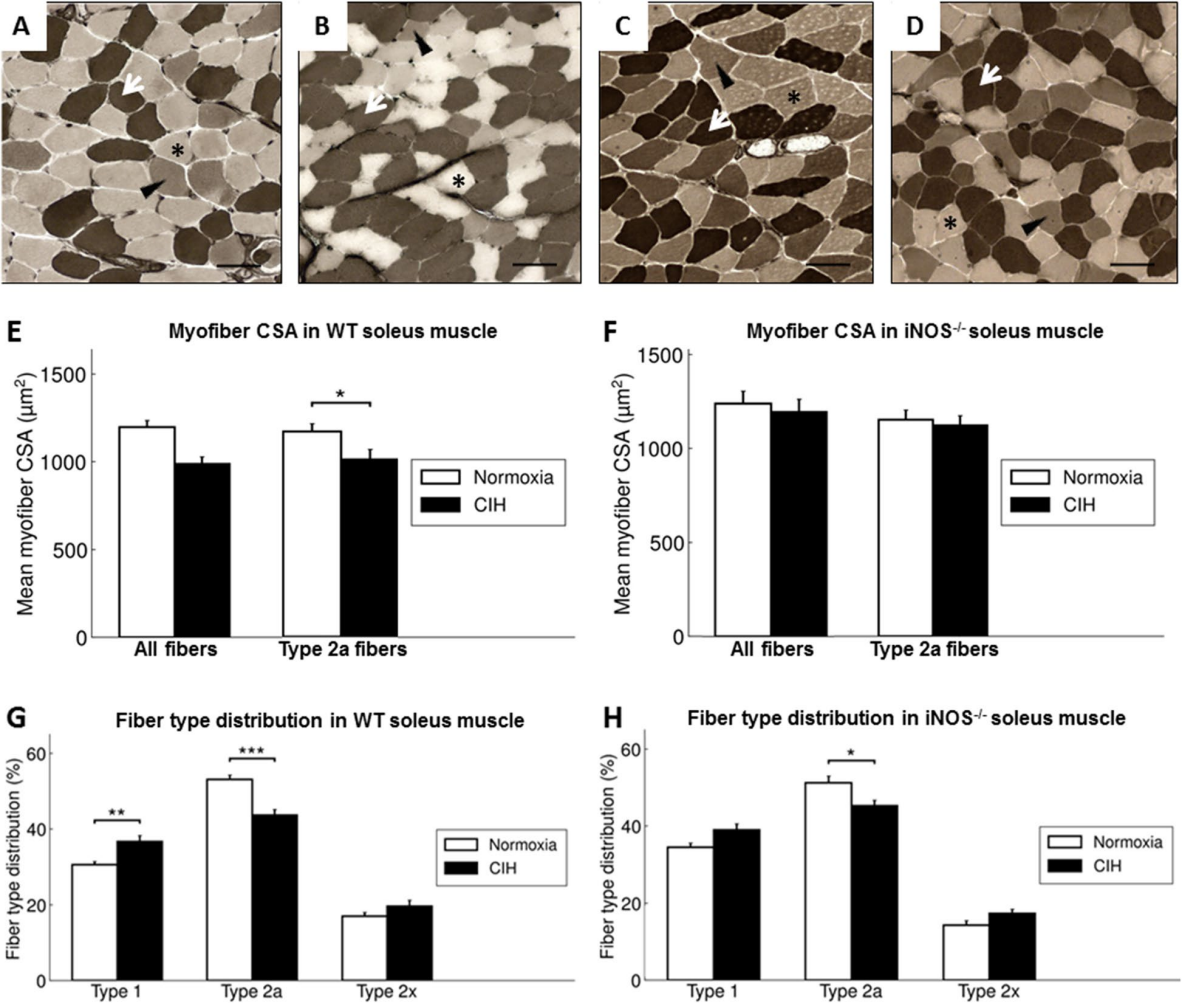
Representative images (200-fold magnification, scale bar = 50 μm) of soleus muscle cross sections stained for ATPase after preincubation at pH 4.55 of WT-NOX mouse (**A**), WT-CIH mouse (**B**), iNOS^−/−^-NOX mouse (**C**), and iNOS^−/−^-CIH mouse (**D**). Type 1 fibers are stained dark (white arrow), type 2a fibers are stained light-colored (black star), and type 2x fibers are stained intermediate (black arrowhead). **E** Overall myofiber CSA [μm^2^] and **F** CSA of type 2a myofibers in the soleus muscle of WT and iNOS^−/−^ mice. Fiber-type distribution [%] in the soleus muscle. Percentage of type 1, type 2a, and type 2x fibers of WT mice after 6 weeks of CIH compared with NOX controls (**G**) and of iNOS^−/−^ mice (H). Values are given as mean + SEM; *n* = 8 to 14 animals per group. *p < 0.05, **p < 0.01, ***p < 0.001, significance between CIH and NOX

**Fig. 2:**
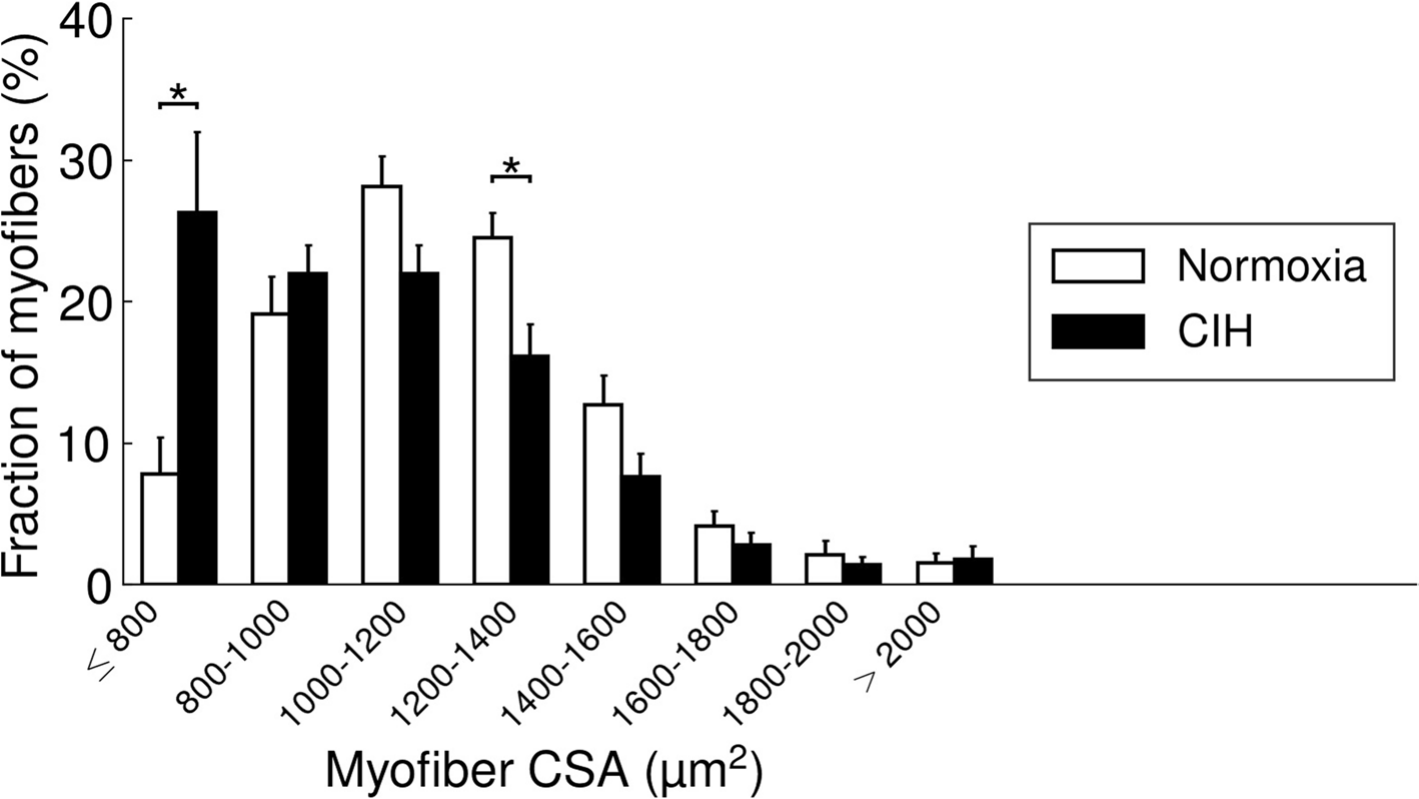
Histogram of myofiber CSA in the soleus muscle in WT mice. Values are given as mean + SEM; *n* = 12 to 14 animals per group. *p < 0.05, significance between WT-CIH and WT-NOX

**Fig. 3:**
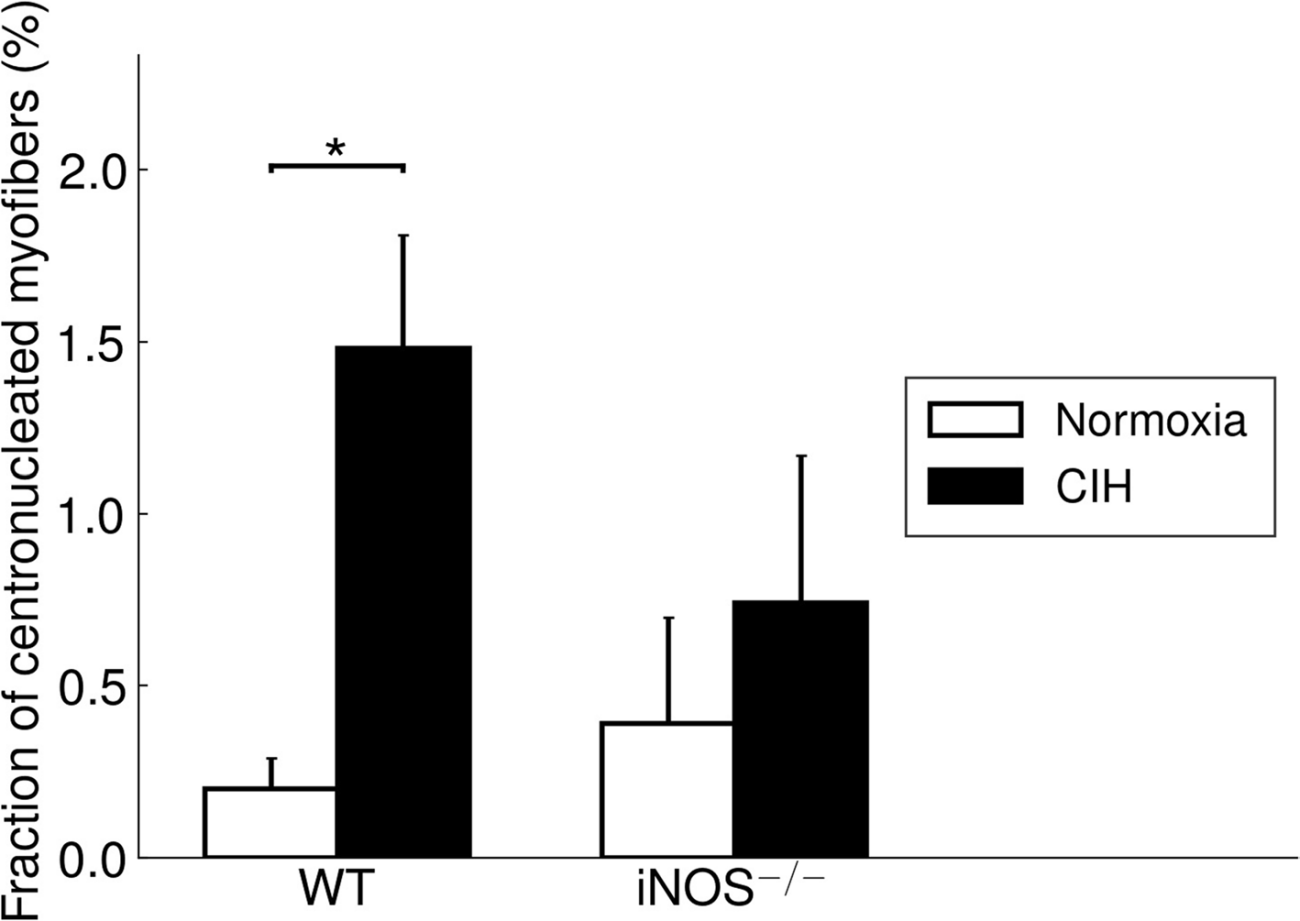
The percentage of centronucleated fibers in the soleus muscle is shown. Values are given as mean+SEM; *n* = 8 to 14 animals per group. *p < 0.05, significance between CIH and NOX

**Fig. 4:**
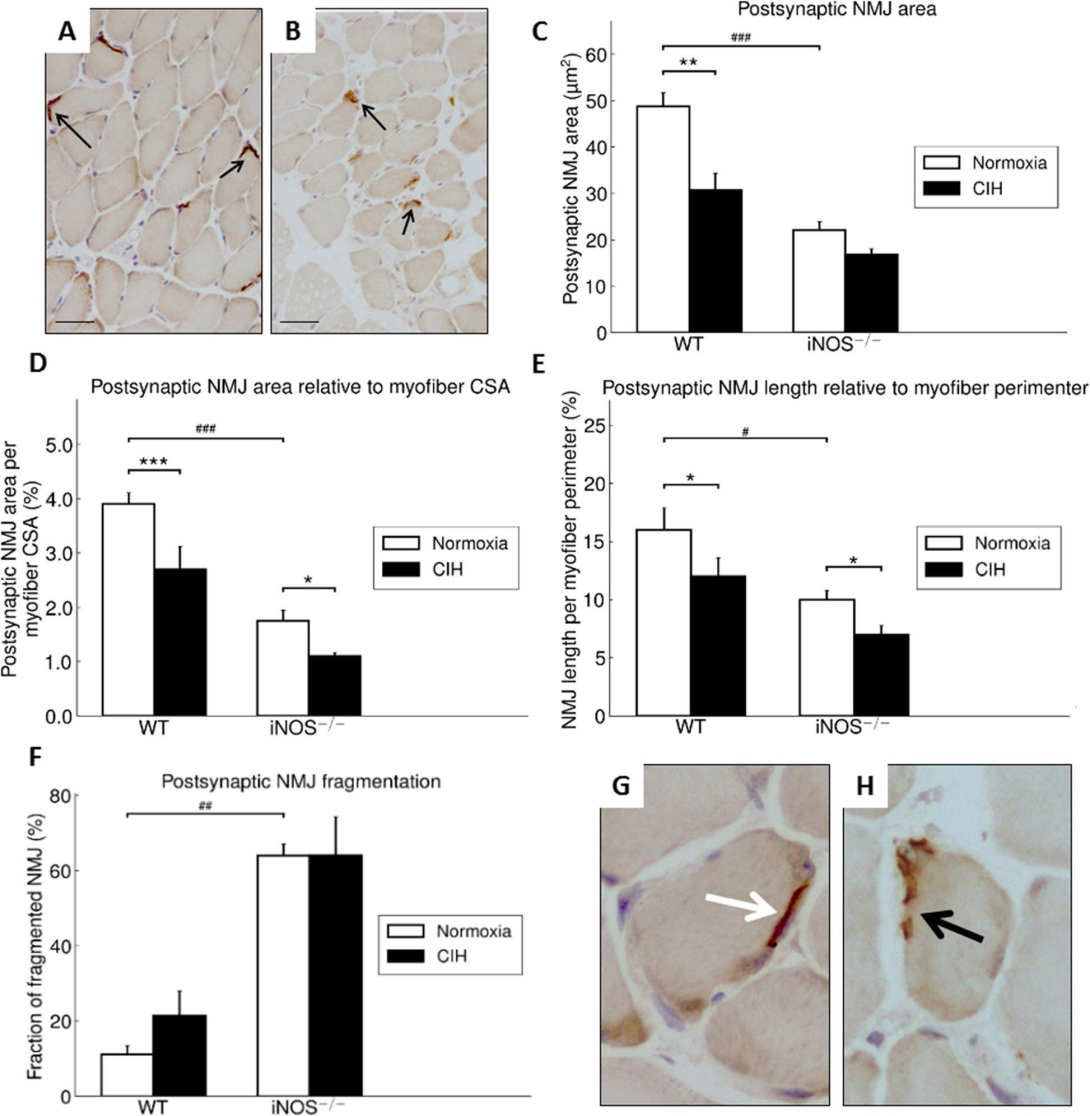
Representative images (200fold magnification, scale bar = 50 μm) of soleus muscle cross sections stained for BTX of WT-NOX (**A**) and WT-CIH (**B**) are shown. Postsynaptic NMJ area in soleus muscle (**C**). Postsynaptic NMJ area normalized to myofiber CSA (**D**) and NMJ length relative to myofiber perimeter (**E**) as well as percentage of fragmented NMJ in soleus muscle (**F**). Representative images (400-fold magnification) of BTX-stained AChR distribution at muscular NMJ in soleus muscle (**G**, **H**). **G** Postsynaptic BTX-stained NMJ from WT-NOX mouse (white arrow). **H** Fragmented postsynaptic BTX-stained NMJ of WT-CIH mouse is (black arrow). Values are given as mean + SEM; *n* = 8 animals per group. *p < 0.05, **p < 0.01, ***p < 0.001, significance between CIH and NOX; ^#^*p* < 0.05, ^##^*p* < 0.01, ^###^*p* < 0.001, significance between WT and iNOS^−/−^

**Fig. 5:**
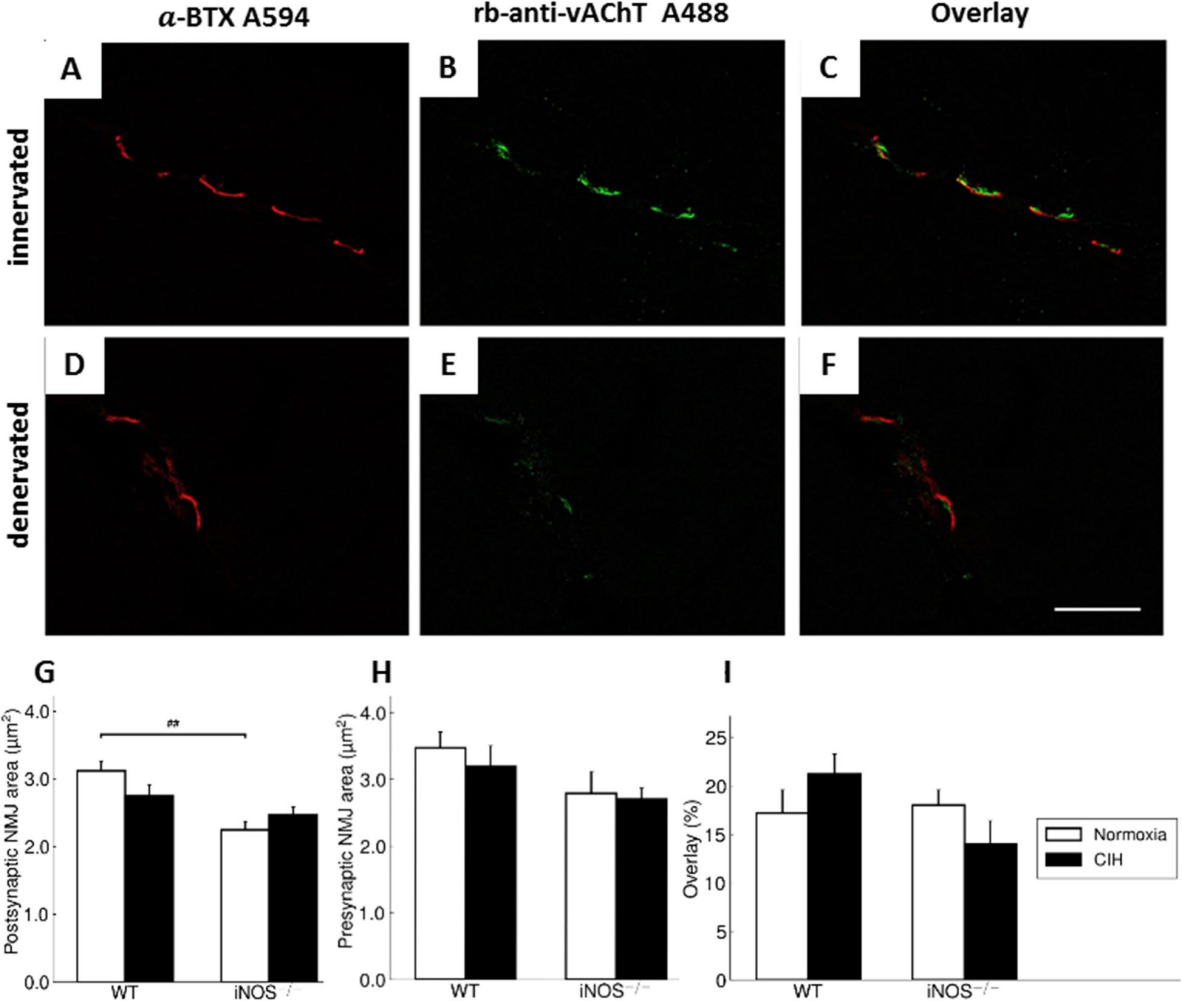
Confocal projection images of representative NMJ from the vastus muscle stained for postsynaptic AChR (BTX, **A** and **D**, red) and for nerve terminal vAChT (**B** and **E**, green). The merge is showing the colocalization of BTX and vAChT (**C** and **F**). Example is given of an innervated muscular NMJ where the BTX-stained area is largely covered by the vAChT staining (**A**–**C**). In contrast, **D**–**F** are showing a NMJ where the AChR-stained areas are only partially covered by vAChT staining, representing denervation. Scale bar in panel **F** is 10 μm. Mean postsynaptic (**G**) and presynaptic (**H**) NMJ areas and overlay areas of BTX and vAChT (**I**) in the vastus muscle are shown. Values are given as mean + SEM; *n* = 4 to 6 animals per group. ^##^*p* < 0.01, significance between WT and iNOS^−/−^

Obstructive sleep apnea (OSA), which describes a repetitive collapse of the upper airways during sleep, causes recurrent episodes of hypopnea or even apnea resulting in chronic intermittent hypoxia (CIH). Over the last two decades, the prevalence of sleep apnea has increased by a double-digit figure [1–5]. In a population-based study among the group of 40- to 85-year-old adults, 49.7% of men and 23.4% of women suffered from a moderate to severe OSA defined as an apnea–hypopnea index (AHI) ≥ 15 [6]. Due to the growing rate of obesity that is suspected as one of the strongest causal triggers, a further increase of sleep-associated disorders is expected [1].

Among various comorbidities, OSA patients often experience muscle fatigue and reduced physical performance [7] resulting in limited daily activities and quality of life. In fact, recent meta-analyses showed that maximum oxygen uptake (aerobic capacity) under cycle ergometer test conditions is significantly reduced in patients with (severe) OSAS [8, 9] compared to healthy controls and may be improved by continuous-positive-airway-pressure (CPAP) therapy [10]. The limiting factors of O_2_ transport or muscle function responsible for an inverse relation between AHI and aerobic capacity have yet remained unclear, but may not include histomorphological muscle microvascularization, which was found to be increased at least in the tibialis anterior muscle [11]. While histomorphological data on locomotor muscle with OSA are surprisingly scarce, several studies in striated upper airway muscles have indicated various important alterations such as fiber-type grouping [12, 13], decreases in fiber cross-sectional area [14], muscle fiber atrophy [12], centrally located nuclei [13], or abnormal mitochondrial distribution [15]. Thus, mechanisms underlying functional impairment of locomotor muscles have remained to be assessed. Notably, adequate muscle function is not only dependent on the integrity of muscle fibers themselves but also reliant on intact neuromuscular signal transmission via myelinated motor neuron, neuromuscular junction (NMJ), and postsynaptic sarcolemma [16]. NMJ integrity and plasticity are considered critical for muscular function: While several studies have demonstrated a relationship between increases in pre- and postsynaptic areas and enhanced neuromuscular activity as well as increased fatigue resistance [17–20], age-related changes in NMJ, such as fragmentation or lowered NMJ area, are considered to contribute to sarcopenia [21, 22]. Moreover, investigations on mice lacking antioxidant enzymes (SOD^−/−^) or overexpressing uncoupling proteins (UCP1) supported the hypothesis, that oxidative stress may trigger NMJ degenerations which occur in association with mitochondrial dysfunction [23, 24]. Most relevant to OSA, a massive generation of reactive oxygen/nitrogen species (ROS/RNS) and related excessive oxidative and nitrosative stress has been attributed to repetitive nocturnal hypoxia-reoxygenation cycles in analogy to repeated ischemia and reperfusion injuries [25, 26]. Indeed, evidence exists in OSA patients for increased production of superoxide, which was normalized under CPAP therapy [27], as well as for a decrease in anti-oxidative capacity [28, 29]. Moreover, studies in OSA patients as well as in CIH animal models detected an overexpression of inducible nitric oxide synthase (iNOS) via inflammatory triggers involving NF-κB activation, especially in neuronal or cardiovascular tissues, e.g., activated macrophages [30–32]. iNOS may become a source of massive amounts of nitric oxide (NO), only limited by a lack of substrate or coenzymes, which as a highly reactive free radical forms peroxynitrite and other RNS compromising mitochondrial respiration, cell membrane integrity or insulin signaling [33]. iNOS expression in skeletal muscle is observed in (type 2 fibers) of obese/diabetic adult, but rarely in healthy young subjects, unless exercising, while muscular iNOS expression in rodents was mainly detectable and studied in rats [34].

The present study used CIH as compared to normoxic control (NOX) as a model of OSA in wildtype (WT) mice to investigate the long-term effect of OSA on skeletal muscle histomorphological measures of NMJ integrity and fiber size and composition as well as on transition electron microscopy (TEM) parameters of ultrastructural mitochondria integrity. Moreover, besides WT, we included iNOS-deficient (iNOS^−/−^) mice into this analysis in order to evaluate the putatively risk-mediating role of iNOS within the pro-oxidative or pro-inflammatory stress arising from CIH exposure. To the best of our knowledge, this is the first report on CIH-induced muscle-specific damage to NMJ, fiber histomorphology, or mitochondrial ultrastructure in WT mice locomotor muscle, which unexpectedly is not inhibited by iNOS deficiency, but strikingly mimicked already at NOX.

## Materials and methods

### CIH mouse model of OSA

The mouse model of OSA was based on the long-term exposure to CIH as described by Schulz et al. (Schulz et al. 2014). Male C57BL/6J-mice (WT, Charles River Deutschland GmbH, Sulzfeld, Germany) and iNOS-deficient mice (iNOS^−/−^; strain B6.129P2-Nos2^tm1Lau^/J) aged 8-10 weeks were exposed to a 6-week CIH profile (8h/day 5 days/week) in a PC-controlled normobaric gas chamber which allowed alternating O_2_ concentration between 7 and 21% at cycles of 120 s corresponding to an AHI of 30h^−1^, i.e., border between moderate to severe sleep apnea. NOX control conditions for age-matched WT and iNOS^−/−^ mice were rendered by the same chamber system flushed with room air (21% O_2_). CIH and control intervention were limited to daytime as mice are active at night. All animals were provided with standard diet and water ad libitum. All mice were weighed prior to intervention and immediately before euthanization.

After 6 weeks of CIH/control intervention, mice were euthanized and the triceps surae (gastrocnemius, soleus, and plantaris muscles) as well as the vastus lateralis muscle carefully removed, immediately shock frozen in liquid nitrogen-cooled isopentane and stored at – 80 °C. Transversal cryosections of 7 μm (microtom Hyrax C60, Carl Zeiss AG, Oberkochen, − 20 °C) were obtained for (immuno)histochemistry. Tibia length was carefully determined by a caliper.

### Muscle fiber composition, size, and centronucleation

Muscle fiber types (1, 2a, 2x) were identified via the acid-sensitive myofibrillar ATPase (adenosine triphosphatase, Sigma-Aldrich Co. LLC, St. Louis, Missouri) staining at pH 4.55 as previously described (Friedmann-Bette et al. 2010) in randomly distributed two to three images taken at 200-fold magnification by the Zeiss Axio Imager.M2 microscope (Carl Zeiss AG; Oberkochen, Germany) after digitalization by the imaging system Axio-Cam HRc/AxioVision (Carl Zeiss GmbH). Type-specific fiber cross-sectional area (CSA) was determined by manually encircling each cross-section of at least 100 fibers using standard imaging software ImageJ (Scion Image, National Institutes of Health, Bethesda, USA). In addition, transverse cryosections were stained by hematoxylin and eosin to determine the percentage of centronucleated fibers. Therefore, each completely imaged fiber was counted manually. In a second step, fibers with centralized nuclei were identified. On the average, 164 +/− 24 fibers were analyzed per muscle.

### Postsynaptic NMJ morphology and integrity

NMJ analyses were based on α-bungarotoxin (BTX) staining in cryosections after fixation with 4% PFA/PBS (paraformaldehyde/phosphate-buffered saline pH 7.4) for 10 min and blocking of endogenous peroxidase with 3% H_2_O_2_. Thereafter, PBS-washed sections were incubated overnight with biotinylated BTX (1:500; Invitrogen Eugene, USA) in a humidified chamber, PBS-washed, blocked with 2% bovine serum albumin (BSA)/PBS, PBS-washed again, and incubated with horseradish peroxidase (HRP)-conjugated streptavidin (Jackson ImmunoResearch Laboratories. Inc., West Grove, USA). 3,3′-Diaminobenzidine (DAB) was used as a chromogen substrate. Nuclei were counterstained with Mayer’s hematoxylin (Carl Roth GmbH, Germany).

NMJ were identified in three consecutive, complete cross-sections per muscle from digital images (200-fold magnification) obtained by the Zeiss Axio Imager.M2 microscope (Carl Zeiss AG; Oberkochen, Germany) combined with Axio-Cam HRc/AxioVision (Carl Zeiss GmbH). The NMJ length and area were determined manually together with the corresponding myofiber CSA and perimeter using ImageJ software (Scion Image, National Institutes of Health). Single NMJ were considered “fragmented” when the BTX-stained area was divided into several sections. Further calculations included NMJ area per myofiber CSA, NMJ length per myofiber perimeter, and the percentage of fragmented NMJ (McLoon et al. 2016). On average, 21 ± 2 (16 ± 3) NMJ were analyzed per soleus or gastrocnemius muscle.

### Immunohistochemistry

For IL1β and CD68 immunohistochemistry, cryostat sections (7 μm) were fixed with 4% PFA/PBS(10 min, ambient temperature). Non-specific sites were blocked with 1% normal swine serum (Dako Deutschland GmbH, Hamburg, Germany) in PBS. Single staining was performed by incubation of polyclonal anti- IL1ß (1:100: Abcam; Cambridge, UK) or monoclonal anti-CD68 primary antibody (1:50; AbD Serotec, Kidlington, UK) with goat polyclonal HRP-conjugated anti-rabbit (1:200; LINARIS GmbH, Dossenheim, Germany) or anti-rat (1:100; AbD Serotec, Kidlington, UK) antibody; endogenous peroxidase activity was suppressed with 3 % H_2_O_2_ in PBS; afterwards, the sections were incubated with DAB solution (Roche Diagnostics). Nuclei were counterstained with Mayer’s hematoxylin (Carl Roth GmbH & Co. KG, Karlsruhe, Germany).

### Pre- and postsynaptic NMJ co-staining

To analyze the innervation status by double staining of NMJ pre- and postsynapse, transverse sections of the soleus (WT: *n* = 8 NOX, *n* = 8 CIH; iNOS^−/−^: *n* = 8 NOX, *n* = 8 CIH) and vastus muscles (WT: *n* = 4 NOX, *n* = 6 CIH; iNOS^−/−^: *n* = 6 NOX, *n* = 6 CIH) were stained for immunofluorescence with biotin-XX-conjugated BTX (Invitrogen Eugene, USA) and RvAChT (vesicular acetylcholine transporter) antibody (Lee Eiden, 80259 Laborchargen-Nr.: bl. 6/97). In detail, after fixation for 10 min in 4% PFA/PBS, and blockage by 1% BSA/PBS for 30min, sections were incubated overnight at 4 °C with biotinylated BTX (1:500) or vAChT 80259 (1:1000) in a humidified chamber. The next day, washing was followed by 2 h of incubation with Alexa Fluor® 488 labeled donkey anti-rabbit IgG (1:200, MoBiTec GmbH, Göttingen, Germany) or Cy3-conjugated streptavidin (1:200, Dianova GmbH, Hamburg, Germany) respectively. Finally, slides were mounted using Immu-Mount™ (Fisher Scientific GmbH, Schwerte, Germany) and glass coverslips. Control sections were treated similarly but with either no primary antibody to exclude non-specific staining of secondary antibody. In the soleus muscle, we additionally included a fourth staining with anti-CD68 antibody (1:50; AbD Serotec, Kidlington, UK), detected by Alexa Fluor® 647 labeled donkey-anti rat IgG (1:200, MoBiTec GmbH, Göttingen, Germany), to visualize macrophages next to the NMJ.

Confocal images were taken by the scanning laser microscope C2 (Nikon GmbH, Düsseldorf, Germany) using the software NIS-Elements AR 4.30.01 (Laboratory Imaging). Each NMJ was scanned in a 630-fold magnification at 250 Hz with an image size of 1024 × 1024 pixels. Subsequently, the Fiji software (National Institute of Health, Bethesda, USA) was used for morphometrical analysis, determining the BTX and vAChT immunolabeled areas as well as their overlap area.

### Transmission electron microscopy

The mitochondrial ultrastructure of the soleus and gastrocnemius muscles was evaluated using TEM as previously described [35]. Images were taken at 10.000-fold magnification and ten random pictures from both muscles of each mouse were analyzed using the ImageJ software (Scion Image, National Institutes of Health, Bethesda, USA). Mean mitochondrial size was determined by manually encircling all identified mitochondria. Alterations on mitochondrial architecture were assessed by the categorization “normal” or “damaged” as follows: Mitochondria showing a loss of more than 50% of the cristae or a disruption of more than 50% of the outer membrane were assigned to “damaged” or otherwise to “normal.” The results were given as the percentage of the total number of mitochondria. Moreover, the percentage of swollen mitochondria and mitochondria containing multi-lamellar bodies was determined.

### RT-qPCR

The extraction of RNA was performed using peqGOLD Isolation Systems TriFast™ (PEQLAB Biotechnologie GmbH, Erlangen, Germany) according to the manufacturer’s instructions. RNA quality (OD260 nm/OD280 nm = 1.7 to 2.0) and concentration were determined using the NanoDrop 2000c spectrophotometer (Thermo Scientific, Schwerte, Germany). RNA integrity was confirmed by lab-on-a-chip technology, using an RNA 6000 NanoChip kit on an Agilent 2100 Bioanalyzer (Agilent Technologies, Waldbronn, Germany). Total RNA (0.7 μg) was then treated with 1 unit DNAse (Thermo Scientific, St. Leon-Rot, Germany; 30 min, 37 °C). Thereafter, reverse transcription of RNA was carried out with 500 ng of oligo (dT)_12–18_ primer, 20 units of the Affinity Script multiple temperature cDNA synthesis (Agilent), and 24 units of Ribo Lock^TM^ RNAse inhibitor (Fermentas; 1 h, 42 °C) and 4 mM dNTP-Mix. Quantitative polymerase chain reaction (qPCR) was performed in duplicates using the QuantiTect Primer Assays from QIAGEN GmbH (Hilden, Germany). To confirm the primer specificity and the presence of a single amplicon, a melting curve (55–95 °C) of the amplified product was performed. For amplification and data analysis, the Mx3005P™ QPCR System (Stratagene) was used. For each sample, the relative amount was calculated by linear regression analysis from their respective standard curves which was generated from a pool of cDNA. Among the transcripts Actb, Gapdh and Tbp, Tbp was identified by the NormFinder software as the most stable reference gene for RNA normalization of the gene of interest (Additional file 1).

### Statistics

Data are presented as means ± standard error of the mean (SEM). Differences between CIH and NOX or iNOS^−/−^ and WT were detected by ANOVA and Student’s *t*-test (post hoc) or, in case of not normally distributed data, by the Kruskal–Wallis ANOVA and Dunn’s post hoc test. A *p* < 0.05 was considered statistically significant. For all statistical procedures, the SigmaPlot 14.0 software (Systat Software, Inc, Chicago, USA) was used.

## Results

### Body weight

#### Effect of CIH in WT

In WT mice, 6 weeks of CIH compared to NOX significantly reduced the pre- to post-interventional gain in body weight, i.e., the weight gain between 2 and 3.5 months of age (starting from a 9.1% higher baseline) (Table 1).

**Table 1:** Weight (g) and weight changes (%) of WT and iNOS^−/−^ mice pre- to post-intervention. Values are given as mean+SEM, *n* = 6 to 14 animals per group. * *p* < 0.05, significance between CIH and NOX; ^##^*p* < 0.01, ^###^*p* < 0.001, significance between WT and iNOS^−/−^

	WT NOX (*n* = 11)	WT CIH (*n* = 14)	iNOS^**−/−**^ NOX (*n* = 6)	iNOS^**−/−**^ CIH (*n* = 8)
Pre-intervention weight (g)	21.9 ± 0.5	23.9 ± 0.5*	24.8 ± 0.7^###^	26.0 ± 0.9
Post-intervention weight (g)	26.4 ± 0.5	25.9 ± 0.3	25.5 ± 0.7	24.1 ± 0.3
Weight change (%)	21.1 ± 3.7	9.0 ± 2.0*	2.9 ± 3.0^##^	− 7.4 ± 0.8*

#### Effect of iNOS w/o CIH

Under NOX conditions, iNOS^−/−^ mice showed a significantly lower weight gain compared to WT (Table 1), notably, starting from by 10.7% (*p* < 0.001) higher pre-interventional body weight than WT mice. When exposed to CIH, iNOS^−/−^ mice even showed a weight loss and thus differed significantly from NOX conditions compared to NOX (Table 1).

### Fiber morphology and fraction in soleus muscle

#### Effect of CIH in WT

The mean CSA covering all fiber types in the soleus muscle did not significantly differ between CIH intervention and NOX in WT (Fig. 1A - B; E, *left*), though it correlated significantly and positively with the weight gain (*r* = 0.41; *p* = 0.047) in WT of both interventional groups. A fiber type-specific analysis showed, that CIH compared to NOX led to a significant decrease in fiber CSA exclusively of type 2a fibers by 13.5% (*p* < 0.05) (Fig 1E, *right*). Thereby, CIH as compared to NOX resulted in an increase in type 1 fiber fraction (37% vs. 31%; *p* < 0.01) in association with a decrease in type 2a fiber fraction (44% vs. 53%; *p* < 0.001) at unchanged type 2x fiber fraction (Fig. 1A - B; G).

A parallel CSA-specific analysis of fiber distribution showed that CIH compared to NOX significantly increased the fraction of small fibers (CSA < 800μm^2^) in the soleus muscle (Fig. 2).

Moreover, there was a significantly higher fraction of centronucleated fibers with CIH compared to NOX (1.5% vs. 0.2%; *p* = 0.01) in WT soleus muscle, as counted in HE-stained sections (Fig. 3, *left*). A significant inverse correlation was found between the fraction of centronucleated fibers and the CSA of the fiber population in WT undergoing CIH or NOX (*r* = − 0.417; *p* = 0.043).

#### Effect of iNOS w/o CIH

In both conditions, NOX and CIH, iNOS^−/−^ compared to WT was without any significant effect on fiber CSA of the total fiber population or that of fiber type 1, 2a, or 2x. In iNOS^−/−^ mice, CIH as compared to NOX caused no significant changes in CSA of fibers in total or of type (Fig. 1F), while CIH-related changes in fiber composition partly resembled those in WT (Fig. 1A, C, H): CIH compared to NOX in iNOS^−/−^ mice led to a decrease in type 2a fiber fraction (45% vs. 51%; *p* < 0.05) and an associated non-significant increase in type 1 fiber fraction (39% vs. 35%) at unchanged type 2x fiber fraction (Fig. 1C, D, H). Concerning the centronucleation, no significant difference was detected between the two genotypes or between CIH and NOX in iNOS^−/−^ mice (Fig. 3).

### Fiber morphology and fraction in the gastrocnemius muscle

In contrast to the soleus muscle, the gastrocnemius muscle mostly consisting of fiber type 2x revealed no significant effect of CIH vs. NOX and iNOS^−/−^ vs. WT or their combination with regard to CSA (Additional file 2) or percentage of central nuclei (Additional file 3).

### NMJ morphology/integrity in soleus muscle

#### Effect of CIH in WT mice

In the soleus muscle of WT mice, BTX staining of NMJ showed that CIH compared to NOX led to a significant 37% smaller postsynaptic NMJ area (Fig. 4A–C, *left*). When calculating NMJ area relative to fiber CSA, (Fig. 4D, *left*), the CIH compared to NOX significantly reduced postsynaptic NMJ size. An alternative normalizing of NMJ length to fiber perimeter similarly decreased NMJ size with CIH compared to NOX (Fig. 4E, *left*). No significant CIH-related change occurred regarding the fraction of fragmented NMJ in WT mice (Fig. 4F, *left*; G).

#### Effect of iNOS^−/−^ w/o CIH

In comparison to WT, iNOS^−/−^ mice demonstrated a significant 55% diminished NMJ area under the condition of NOX (Fig. 4C), with this effect being significant also upon normalization of postsynaptic NMJ area for fiber CSA (Fig. 4D) or, alternatively, of postsynaptic NMJ length for fiber perimeter (Fig. 4E). CIH compared to NOX intervention in iNOS^−/−^ mice led to an additional decrease in NMJ area (in absolute terms) by trend (Fig. 4C, *right*); however, this effect reached significance when normalizing postsynaptic NMJ area for fiber CSA (Fig. 4D, *right*) or, alternatively, postsynaptic NMJ length for fiber perimeter (Fig. 4E, *right*). Notably, a strikingly higher percentage of NMJ fragmentation was observed selectively with iNOS^−/−^ as compared to WT under both conditions (Fig. 4F, *right*; H).

### NMJ morphology/integrity in gastrocnemius and vastus muscles

#### Gastrocnemius muscle

In contrast to the soleus muscle, the gastrocnemius muscle in WT mice showed a significantly increased NMJ size with CIH as compared to NOX (72%, *p* < 0.05, Additional file 4). However, this difference was abolished when normalizing postsynaptic NMR area for fiber CSA. Moreover, unlike the soleus muscle, the gastrocnemius muscle in iNOS^−/−^ mice revealed no significant alterations in postsynaptic NMJ area, and this was also true for both abovementioned normalization of NMJ area or length for fiber CSA or perimeter, respectively.

#### Vastus muscle

For further evaluation of functional NMJ integrity, double fluorescent staining was used in the vastus muscle to quantify the area of the NMJ presynaptic nerve terminal (anti-vACHhT-antibodies, Fig. 5A, D), of postsynaptic NMJ (BTX, Fig. 5B, E) and that of their coupling (overlay, Fig. 5C, F). In line with the findings in the soleus (but not in gastrocnemius) muscle, there was a significant diminution by 27.9% (*p* < 0.01) of postsynaptic NMJ in iNOS^−/−^ compared to WT under conditions of NOX (Fig. 5G), whereas CIH effects compared to NOX were absent in WT or iNOS^−/−^ mice. The presynaptic terminal, defined as the vAChT immunoreactive area, remained resistant against CIH- or genotype-related effects (5H). The resulting percentage overlay area, a measure of NMJ integrity, was not significantly affected by CIH intervention or iNOS^−/−^ (Fig. 5I).

#### Inflammatory markers (IL1β, CD68) in soleus and gastrocnemius muscles

**Fig. 6:**
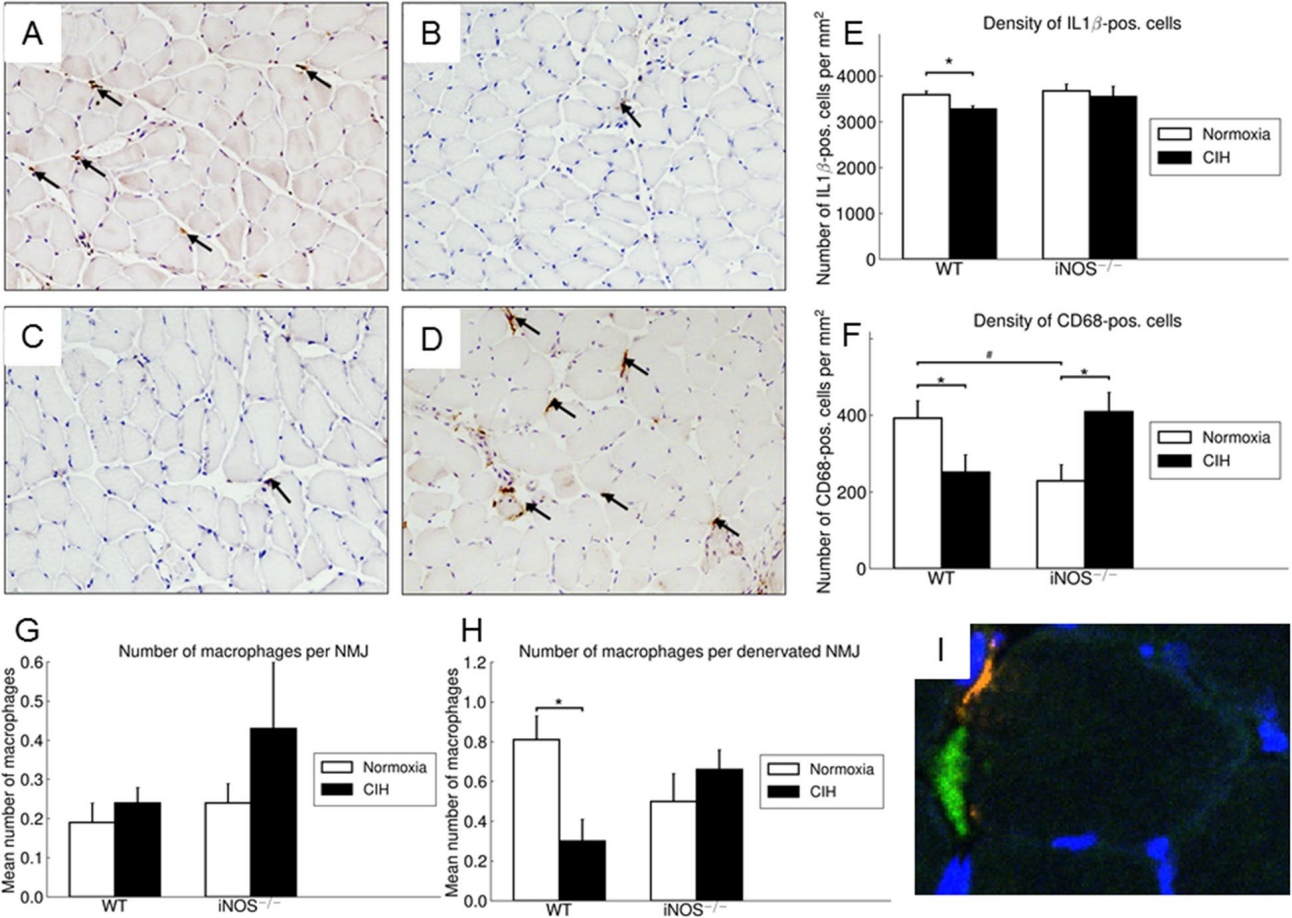
Representative images (200fold magnification, scale bar = 50 μm) of soleus muscle cross sections stained for CD68 of WT-NOX (**A**), WT-CIH (**B**), iNOS^−/−^-NOX (**C**), and iNOS^−/−^-CIH (**D**) are shown. CD68-positive cells are marked with arrows. Density of IL 1β- (**E**) and CD68- (**F**) positive cells in the soleus muscle is shown. Mean number of macrophages per NMJ (**G**) and mean number of macrophages per denervated NMJ (**H**). Merge of confocal projection images of representative NMJ from the soleus muscle stained for nerve terminal vAChT (green) and CD68 (red) showing the colocalization of NMJ and macrophages (**H**). Values are given as mean + SEM, *n* = 8 animals per group. * *p* < 0.05, significance between CIH and NOX, ^#^*p* < 0.05, significance between WT and iNOS^−/−^

In WT soleus muscle, a significantly almost 9% reduced density in IL 1ß-positive cells was found under CIH compared to NOX (*p* < 0.05) (Fig. 6E) and iNOS^−/−^ compared to WT, as well as iNOS^−/−^ CIH vs. NOX was without any significant effect (Fig. 6E). In the gastrocnemius, neither CIH- nor iNOS^−/−^-related changes were detected.

Analyzing the status of interstitial macrophages (CD68-positive cells), sections of WT mice soleus displayed a significant 36% reduction of macrophage density in CIH vs. NOX (Fig. 6A, B, F) while no such differences were found in the gastrocnemius muscle. In comparison to WT, iNOS^−/−^ mice demonstrated a significant 78% increased CD68 density under the condition of CIH compared to NOX (Fig. 6C, D, F). Furthermore, a significantly diminished density was observed with iNOS^−/−^ as compared to WT under NOX (*p* < 0.05) (Fig. 6A, C, F).

Given that macrophages are reported to be present at the NMJ after nerve injury, we studied the amount of macrophages next to the intact as well as damaged NMJ in the soleus muscle using double fluorescent staining (Fig. 6I). NMJ were considered denervated when the overlay of BTX and vAChT staining was less than 10%.

The mean number of macrophages covering all NMJ in soleus muscle did not significantly differ between CIH intervention and NOX in WT or iNOS^−/−^ (Fig. 6G). A specific analysis of denervated NMJ showed, that CIH compared to NOX led to a significant decrease of macrophages next to the endplate (0.30 vs. 0.81; *p* < 0.02) (Fig. 6H).

Furthermore the macrophage density in the soleus muscle correlated significantly and positively with the overlay area of BTX and vAChT (*r* = 0.55; *p* = 0.004) as well as negatively with the amount of denervated NMJ (*r* = − 0.59; *p* = 0.001).

#### Mitochondrial ultrastructure in soleus muscle

**Fig. 7:**
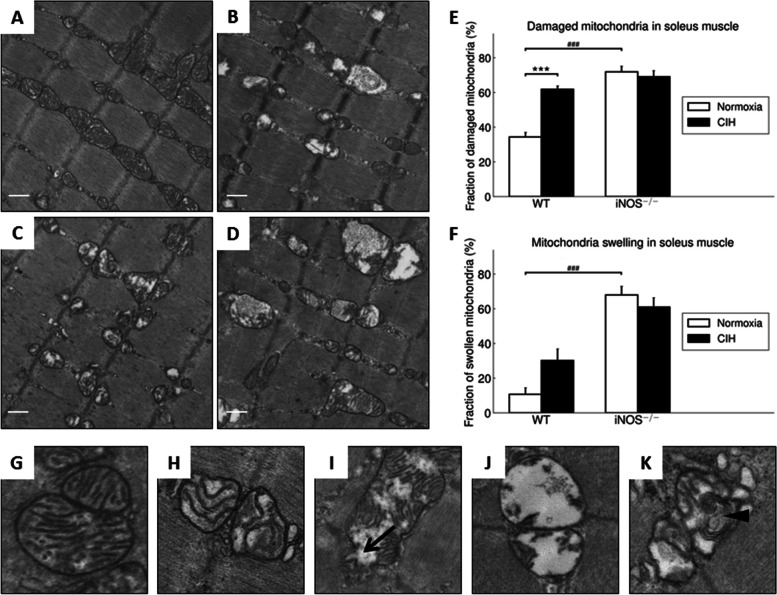
Representative TEM images of WT (**A**, **B**) and iNOS^−/−^ (**C**, **D**) soleus muscle under CIH (**B**, **D**) vs. NOX (**A**, **C**) (scale bars = 100 nm). Percentage of damaged (**E**) and swollen mitochondria (**F**) are shown. Mitochondria presented various morphological abnormalities such as outer membrane rupture (**I**; black arrow), loss of cristae (**J**), or multi-lammellar bodies (**K**; black arrowhead). **G** and **H** correspond to intact mitochondria completely (**G**) or partially (**H**) filled with cristae. Values are given as mean + SEM, *n* = 7 to 9 animals per group. ***p < 0.001, significance between CIH and NOX, ^###^*p* < 0.001, significance between WT and iNOS^−/−^

The following ultrastructural mitochondrial abnormalities were quantified in soleus muscle of WT exposed to NOX (Fig. 7A) or CIH (Fig. 7B) as well as in iNOS^−/−^ mice in NOX (Fig. 7C) or CIH (Fig. 7D): Swollen matrix (Fig. 7H), disruption of the outer mitochondrial membrane (Fig. 7I), a complete loss of internal architecture (Fig. 7J) and mitochondria with multi-lamellar bodies (Fig. 7K).

#### Effect of CIH in WT

The percentage of damaged mitochondria, classified as <50% filled with cristae, was significantly 1.8-fold higher in CIH vs. NOX (Fig. 7E, *left*), while an increase in the percentage of swollen mitochondria with CIH did not reach significance (Fig. 7F, *left*). The difference between CIH vs. NOX regarding the percentage of mitochondria containing multi-lamellar bodies did not reach significance (12.8% vs. 6.6%; *p* > 0.05).

#### Effect of iNOS^−/−^ w/o CIH

Somewhat reminiscent of CIH vs. NOX effects in WT, iNOS^−/−^ compared to WT mice at NOX conditions revealed a significant 2.1-fold increase of damaged mitochondria (Fig. 7E). Moreover, the percentage of swollen mitochondria in iNOS^−/−^ vs. WT mice was significantly increased under both conditions (NOX > 6-fold; CIH > 5-fold) (Fig. 7F).

#### Mitochondrial ultrastructure in the gastrocnemius muscle

In the gastrocnemius muscle, the abovementioned mitochondrial alterations were observed neither with CIH vs. NOX in WT nor with iNOS^−/−^ of either condition (Additional files 5 and 6).

#### Correlations between NMJ fiber and mitochondrial morphology

In the soleus muscle, the NMJ area correlated significantly with the percentage of damaged mitochondria (*r* = 0.584, *p* = 0.002) as well as with the ratio between type 1 and type 2a fibers (*r* = 0.397, *p* = 0.05) (Table 2).

**Table 2:** Correlations of NMJ area and fragmented NMJ, percentage of damaged mitochondria, myofiber CSA, and fiber type ratio (1/2a) in soleus muscles, *n* = 5 to 8 animals per group

Soleus muscle (*n* = 5 to 8 animals per group)				
		NMJ area (μm^2^)	Fragmented NMJ (%)	Fiber-type ratio; 1/2a
Damaged mitochondria (%)	*r*	− 0.710	0.584	0.394
	*p*	0.000	0.002	0.051
NMJ area (μm^2^)	*r*		− 0.642	− 0.575
	*p*		0.001	0.003
Fragmented NMJ (%)	*r*			0.396
	*p*			0.050

#### Transcripts of iNOS, SOCS3, IL6, SOD2, and pro-/anti-apoptotic markers

Notably, mRNA expression of iNOS was undetectable in soleus (as well as in gastrocnemius) muscle of WT mice undergoing NOX or CIH intervention (Table 3). However, importantly, an iNOS expression was well detectable in the liver of WT mice, where it decreased significantly and massively by factor 0.12 (Table 3) after CIH-treatment. The absence of iNOS expression was proven in the liver and soleus (and gastrocnemius) muscle of iNOS^−/−^ mice.

**Table 3:** Relative gene expression in soleus muscle and liver of WT and iNOS^**−/−**^ mice

	iNOS	SOCS3	SOD2	BAX	BCL2	Caspase 3	IL6
**Soleus muscle**							
WT NOX	0.00	1.00	1.00	1.00	1.00	1.00	1.00
WT CIH	0.00	10.89	0.53	1.10	1.14	0.90	0.98
iNOS^−/−^ NOX	0.00	10.45	0.75	1.23	1.01	1.09	0.79
iNOS^−/−^ CIH	0.00	4.43	0.55	0.98	1.07	0.79	1.41
**Liver**							
WT NOX	1.00						
WT CIH	0.12						

Importantly, in the soleus muscle of WT mice, SOCS3 expression was found to be >10-fold increased with CIH compared to NOX (Table 3). Similarly, iNOS^−/−^ prompted a SOCS3 upregulation that was >10-fold in NOX and >4-fold in CIH as compared to WT at NOX (Table 3). Notably, mRNA expression of SOCS3 was undetectable in the gastrocnemius of WT mice. Comparing iNOS^−/−^ CIH and NOX, SOCS3 increased by factor 2.8.

IL6 as an upstream factor of SOCS3 showed neither CIH- nor iNOS^−/−^-related changes in soleus muscle (Table 3). In WT mice gastrocnemius muscle, CIH compared to NOX led to a 50% decrease of IL6 mRNA expression while in iNOS^−/−^ no relevant difference between CIH and NOX was found.

Moreover, in WT mice, CIH compared to NOX led to an almost 50% decrease in mtSOD mRNA expression in soleus muscle. Similarly, iNOS^−/−^ resulted in a 33% and a 45% decrease in SOD2 expression in soleus with NOX and CIH, respectively (Table 3).

Furthermore, the screening for apoptotic markers (BAX, BCL2, caspase 3) showed neither CIH- nor iNOS^−/−^-related changes in soleus muscle (Table 3).

## Discussion

Using long-term CIH exposure in mice as a model of OSA, the present study shows for the first time that CIH compared to NOX causes damage of potential functional relevance in “red” (soleus) but not in “white” (gastrocnemius) muscle. This comprises a reduction in area, in length, and, by trend, in integrity of postsynaptic NMJ as well as in size (CSA) and fraction of type 2a fibers (at higher type-1 fiber fraction). Moreover, these changes were associated with considerable mitochondrial damage, which showed a significant correlation to (loss in) NMJ area (*r* = − 0.71, *p* < 0.001) and were, again, limited to the soleus muscle, while the gastrocnemius revealed no significant mitochondrial damage.

The present study furthermore included iNOS^−/−^ mice into this analysis of CIH vs. NOX effects on skeletal muscle, in order to test the hypothesis that iNOS deficiency may at least in part protect against a pro-inflammatory/pro-oxidative effect through CIH, i.e., hypoxia-reoxygenation stress leading to ROS generation from various sources [36]. Contrary to expectation, our data demonstrate that, compared to WT, iNOS^−/−^ by itself (i.e., under NOX conditions) also leads to highly significant postsynaptic NMJ area reduction and fragmentation in combination with mitochondrial damage and swelling, which surprisingly resemble and exceed those observed with CIH in WT mice. Notably, under the conditions of iNOS deficiency, CIH stress is able to further aggravate the damage at least in terms of a further reduction in postsynaptic NMJ area or length after normalization for fiber CSA or perimeter, respectively. The similarity between CIH (compared to NOX in WT) and iNOS^−/−^ (compared to WT in NOX) was limited to NMJ and mitochondrial damage, while decreases in fiber CSA (including its correlation to NMJ) and centronucleation observed with CIH vs. NOX in WT were absent in iNOS^−/−^ mice, i.e., they revealed no atrophy despite signs of denervation.

As another striking similarity, we found a > 10-fold increase in SOCS3 expression with CIH vs. NOX in WT as well as with iNOS^−/−^ vs. WT at NOX in (pooled samples of) soleus muscle. Available evidence qualifies SOCS3 as a candidate to mechanistically link mitochondrial damage to NMJ deterioration: SOCS3 upregulation has been demonstrated as an early event after skeletal muscle denervation by sciatic nerve transection [37].

SOCS3 overexpression has been shown to cause mitochondrial damage like swelling or disruption in the tibialis anterior muscle, which is reminiscent of what was presently observed in the soleus muscle but not in the gastrocnemius muscle. SOCS3 overexpression was, furthermore, associated with inhibited expression of mitochondrial genes, which included Smtck and Slc25a3 [38], but may also comprise mtSOD, which was presently found to be downregulated. As an inhibitor of leptin and insulin signaling, increased muscular SOCS3 expression has been suggested as a major contributor to mitochondrial dysfunction, impaired fatty acid oxidation, as associated with aging, metabolic syndrome, and inflammation [39–41]. These severe metabolic effects in combination with previous evidence that SOCS3 overexpression dilates the sarcoplasmic reticulum, dislocates and inhibits calcineurine (colocalized with SOCS3), and reduces skeletal muscle energy expenditure, oxygen uptake, and activity [38] may contribute to muscle fiber atrophy as observed in case of CIH. While we found no evidence for increased apoptosis signals, a SOCS3 upregulation appears to be associated with impaired regenerative stem cell function in elderly humans [41] and may potentially play a role in the increased centronucleation presently observed with CIH.

Althoug it is assumed that SOCS3 upregulation occurs in response to local inflammatory signals, especially via IL6, in this study, no difference in IL6 expression under CIH exposure was found. Instead, we even observed a lower level of inflammatory markers like Il1β and a reduced macrophage density in the soleus but not the gastrocnemius muscle. Furthermore, in the soleus muscle, CIH-exposed WT mice compared to normoxic controls showed a reduced amount of macrophages next to denervated NMJ while there was no difference regarding the intact NMJ. Previous studies gave evidence that macrophages play a beneficial role in reinnervation at the NMJ [42], so that the observed loss of macrophages at the denervated NMJ under CIH exposure might indicate a lower regenerative capacity of damaged NMJ. Moreover, loss of macrophages in skeletal muscles is described in spinal muscular atrophy mice as the disease becomes worse which correlates with the disruption of NMJ [43, 44]. As observed in our study, the disruption of NMJ under the condition of SMA is more severe in slow-twitch muscles than in fast-twitch muscles [44].

Moreover, the moderate decrease in mtSOD expression presently observed in soleus muscle with CIH and, to a lesser extent, with iNOS (NOX or CIH) might also play a role in fiber atrophy and mitochondrial deterioration: *Sod1*^−/−^ mice, used as a murine model of neuromuscular impairment in age-related muscle atrophy (sarcopenia), exhibit reduction in myofiber CSA of type IIa fibers [45, 46]. Reduced CSA of type IIa fibers was, indeed, presently observed in association with the most marked mtSOD decrease (approx. 50%), lower weight gain, and alterations in mitochondrial ultrastructure and NMJ morphology. In addition, the observed shift in fiber metabolic phenotype, i.e., an increased type 1 at a decreased type 2 fiber fraction with both CIH (vs. WT) and iNOS^−/−^ (vs. WT at NOX) might be attributed to decreased mtSOD expression, rather than to SOCS3 upregulation which decreases oxidative fiber characteristics [38]. Deficiency in mtSOD, representing impaired antioxidant defense, may also be involved in a remarkable number of age-related features which may originate from a loss of fast motoneurons followed by a reinnervation of slow motoneurons [47]. In the present study, fiber-type ratio (type 1/type 2a) was significantly correlated with NMJ fragmentation and inversely correlated with NMJ size, pointing at a role of reinnervation in the fiber type shift.

Thus, our observations with CIH may display some analogies to age-related neuromuscular deterioration involving SOCS3 and mtSOD. They are in line with previous studies in other rodent CIH models, revealing downregulation of mtSOD/SOD2 via downregulation of HIF-2α [48] and clinical observation of lower plasma CuZnSOD/SOD1 in OSA patients [49].

Our CIH-based OSA mouse model is, however, at variance with biopsy studies in OSA patients, which revealed no changes in tibialis anterior muscle fiber size compared to controls [11] or showed even enlarged diameters of type 2a fibers in the quadriceps femoris at unaltered fiber-type composition [50]. One should, however, bear in mind, that the CIH mouse model does not mimic certain OSA inherent factors like sways in intrathoracic pressure and blood pCO_2_ as well as ventilatory overshoots but at the same time involves more severe O_2_ desaturation without airway obstruction. Also, the genetic background of mice may affect the degree of atrophy [51].

Even more important, our data provide first evidence for a strikingly differential effect of CIH-exposure between soleus and gastrocnemius, i.e., (mixed) “red” and “white” muscles, that has to be taken into account in translational studies. Indeed, in contrast to the soleus muscle, the gastrocnemius muscle revealed neither mitochondrial damage nor NMJ alterations (rather enlargement than shrinkage) with CIH. The differential exertion profiles between the postural soleus muscle (remaining recruited throughout during quiet standing) and the locomotor gastrocnemius muscle (providing fast forceful contractions) [52] may impact these muscle-specific findings in CIH and iNOS^−/−^ mice and have likewise been implicated in massive muscle- (fiber-) specific differences in muscle aging or neurodegenerative disease [53–55].

However, our findings of compromised mitochondrial ultrastructure and gene expression in soleus muscle of CIH-mice may be in line with those in human palate muscle (a primary research focus within OSA pathophysiological), showing abnormal mitochondrial function and organization [56]. The observed close positive correlation of the fraction of damaged mitochondria to fragmented NMJ (or inverse correlation to NMJ area) reveals no clue for cause–effect relationship. As a first assumption, mitochondria-derived oxidative stress during CIH (hypoxia–reoxygenation stress) may compromise NMJ [57] acting in combination with other ROS sources like upregulated NOX2, as reported for the presently used CIH mouse model [58]. However, importantly, iNOS^−/−^ at NOX largely mimicked the CIH effects, i.e., NMJ and mitochondrial damage together with SOCS3 upregulation and mtSOD downregulation and was despite the fact that iNOS mRNA expression in soleus or gastrocnemius muscles in WT was neither detectable with NOX nor with CIH exposure. Since, in contrast, WT mice revealed a hepatic iNOS expression, which was massively and significantly reduced (> 8-fold) with CIH compared to NOX, it is reasonable to assume that iNOS deficiency outside the skeletal muscle conveys both, the CIH and iNOS^−/−^ effects. Thereby the NMJ damage, similarly observed with both these conditions, strongly points towards an iNOS deficiency in peripheral nerves (i.e., in perikaryon of motoneurons or Schwann cells) as a cause of NMJ damage, though myeloid iNOS expression may also become muscle-protective [59]. Peripheral nerve injury may dramatically upregulate the low constitutive iNOS expression in Schwann cells, and iNOS deletion may result in smaller regenerating myelinated fibers and delayed reinnervation of muscle NMJ distal to the injury [60]. In fact, peripheral nerve dysfunction in patients suffering from OSA appears to be an early event [61], and denervation may precede muscular dysfunction, as suggested for human upper airway muscles [37, 62, 63] and supported by increased sarcolemmal N-CAM staining [15]. To date, no corresponding neuromuscular data exist for human locomotor muscles with OSA. However, they are needed to evaluate the functional relevance of these alterations and to separate OSA-specific effects on NMJ, mitochondria, metabolism, and related fiber dysfunction from the processes of aging and degenerative diseases [21, 64, 65]. Notably, the age of mice presently under test (four months) corresponded to early human adulthood (20–30 years) [66].

The conclusion that iNOS expression (outside skeletal muscle) may be neuro-protective and relevant for “red” (aerobic) muscle function may be somewhat counterintuitive, as iNOS upregulation is resulting in a boost of NO, that is antimicrobial or antitumoral but also cytotoxic to normal tissue [67]. Furthermore it is causally implicated, e.g., in insulin resistance and diabetes. Nonetheless, a basal NO production rate (rendered mostly but not exclusively by nNOS and eNOS) is physiologically required in humans (reviewed by [34]). There is evidence that NO may convey physiological oxidative signals [68] and a certain production by iNOS is required for neuroprotective antioxidative defense [69], e.g., through the ROS scavenging function of NO [67, 69]. Data on skeletal muscle tissue are scarce; however, it was reported that iNOS deficiency leads to mitochondrial damage in myocardial dysfunction (adriamycin-based mouse model). Interestingly, this effect was abrogated by overexpression of mtSOD [70, 71], which presently was found to be downregulated with both CIH and iNOS deletion.

As a limitation, this study includes no functional data regarding NMJ and skeletal muscle to challenge the relevance of morphological alterations. Moreover, our mouse model involved a limited CIH exposition of 5 days per week, which may allow adaptive or protective effects of 2 normoxic days per week. Nonetheless, a previous study showed that the pathophysiological changes of the clinical OSA, such as arterial hypertension, are accurately reflected by the here used CIH mouse model [58].

## Conclusion

In summary, this is the first study to demonstrate that CIH as a model of moderate to severe OSA triggers NMJ and mitochondrial damage accompanied by fiber atrophy in the slow-twitch muscle of WT mice, all of which may contribute to reduced exercise (aerobic) capacity in patients suffering from OSA. We furthermore demonstrate that iNOS deficiency, rather than yielding protection of skeletal muscle against CIH stress, leads to similar structural impairments of NMJ and mitochondria under normoxia and might contribute to the CIH effects in WT, putatively through compromised innervation.

## Supplementary Information

**Additional file 1.** Primers used for real time RT-qPCR.

**Additional file 2. **The myofiber CSA in gastrocnemius muscle is shown. Values are given as mean+SEM; *n* = 8 to 10 animals per group.

**Additional file 3. **The percentage of centronucleated fibers in gastrocnemius muscle is shown. Values are given as mean+SEM; *n* = 8 to 10 animals per group.

**Additional file 4. **The post-synaptic NMJ area in gastrocnemius muscle is shown. Values are given as mean+SEM; *n* = 8 to 10 animals per group. * *p*<0.05, significance between CIH and NOX.

**Additional file 5. **The percentage of damaged mitochondria in gastrocnemius muscle is shown. Values are given as mean+SEM; *n* = 8 to 10 animals per group.

**Additional file 6. **The percentage of swollen mitochondria in gastrocnemius muscle is shown. Values are given as mean+SEM; *n* = 8  to 10 animals per group.

## Data Availability

The datasets used and/or analyzed during the current study are available from the corresponding author on reasonable request. Materials used in this study are commercially available.

## Abbreviations

OSA: Obstructive sleep apnea
CIH: Chronic intermittent hypoxia
NMJ: Neuromuscular junction
NOX: Normoxia
CSA: Cross-sectional area
ATP: Adenosine triphosphate
TEM: Transmission electron microscopy
WT: Wildtype
iNOS: Inducible nitric oxide synthase
SOCS3: Suppressor of cytokine signaling 3
AHI: Apnea–hypopnea index
CPAP: Continuous positive airway pressure
UCP: Uncoupling protein
ROS: Reactive oxygen species
RNS: Reactive nitrogen species
NO: Nitric oxide
BTX: α-Bungarotoxin
PFA: Paraformaldehyde
PBS: Phosphate-buffered saline
HRP: Horseradish peroxidase
BSA: Bovine serum albumin
DAB: 3,3′-Diaminobenzidine
vAChT: Vesicular acetylcholine transporter
Actb: Actin beta
GADPH: Glyceraldehyde 3-phosphate dehydrogenase
TBP: TATA-box-binding protein
SEM: Standard error of the mean
mtSOD: Mitochondrial superdioxide dismutase
BAX: BCL-2-associated X protein
BCL2: *B* cell lymphoma 2
MURF-1: Muscle RING-finger protein-1
sMtCK: Sarcomeric mitochondrial creatine kinase
Slc25a3: Solute carrier family 25 member 3
nNOS: Neuronal nitric oxide synthase
eNOS: Endothelial nitric oxide synthase
