# Supplementary material for: Effect of chronic intermittent hypoxia (CIH) on neuromuscular junctions and mitochondria in slow- and fast-twitch skeletal muscles of mice—the role of iNOS
Source: Skelet Muscle. 2022 Feb 12;12:6. doi: 10.1186/s13395-022-00288-7 (PMC8841105; doi:10.1186/s13395-022-00288-7)
Supplement: Supplementary file 1 — Additional file 1. Primers used for real time RT-qPCR. [file 13395_2022_288_MOESM1_ESM.docx]

| **Primer** | **Symbol** | **Amplicon Length (bp)** | **Cat. no.** |
| --- | --- | --- | --- |
| **Genes of interest** | | | |
| B cell leukemia/lymphoma 2 | Bcl2 | 104 | QT02392292 |
| BCL2-associated X protein | Bax | 78 | QT00102536 |
| Caspase 3 | Casp3 | 150 | QT01164779 |
| Interleukin 6 | IL6 | 128 | QT00098875 |
| Nitric oxide synthase 2, inducible | iNOS | 118 | QT00100275 |
| Superoxide dismutase 2, mitochondrial | mtSOD | 159 | QT00161707 |
| Suppressor of cytokine signaling 3 | Socs3 | 90 | QT02488990 |
| **Reference genes** | | | |
| Actin, beta | Actb | 77 | QT01136772 |
| Glyceraldehyde-3-phosphate dehydrogenase | Gapdh | 144 | QT01658692 |
| TATA box binding protein | Tbp | 114 | QT00198443 |

**Additional file 1: Primers used for real time qRT-PCR.**
