# Supplementary figures and images for: Effect of chronic intermittent hypoxia (CIH) on neuromuscular junctions and mitochondria in slow- and fast-twitch skeletal muscles of mice—the role of iNOS
Source: Skelet Muscle. 2022 Feb 12;12:6. doi: 10.1186/s13395-022-00288-7 (PMC8841105; doi:10.1186/s13395-022-00288-7)

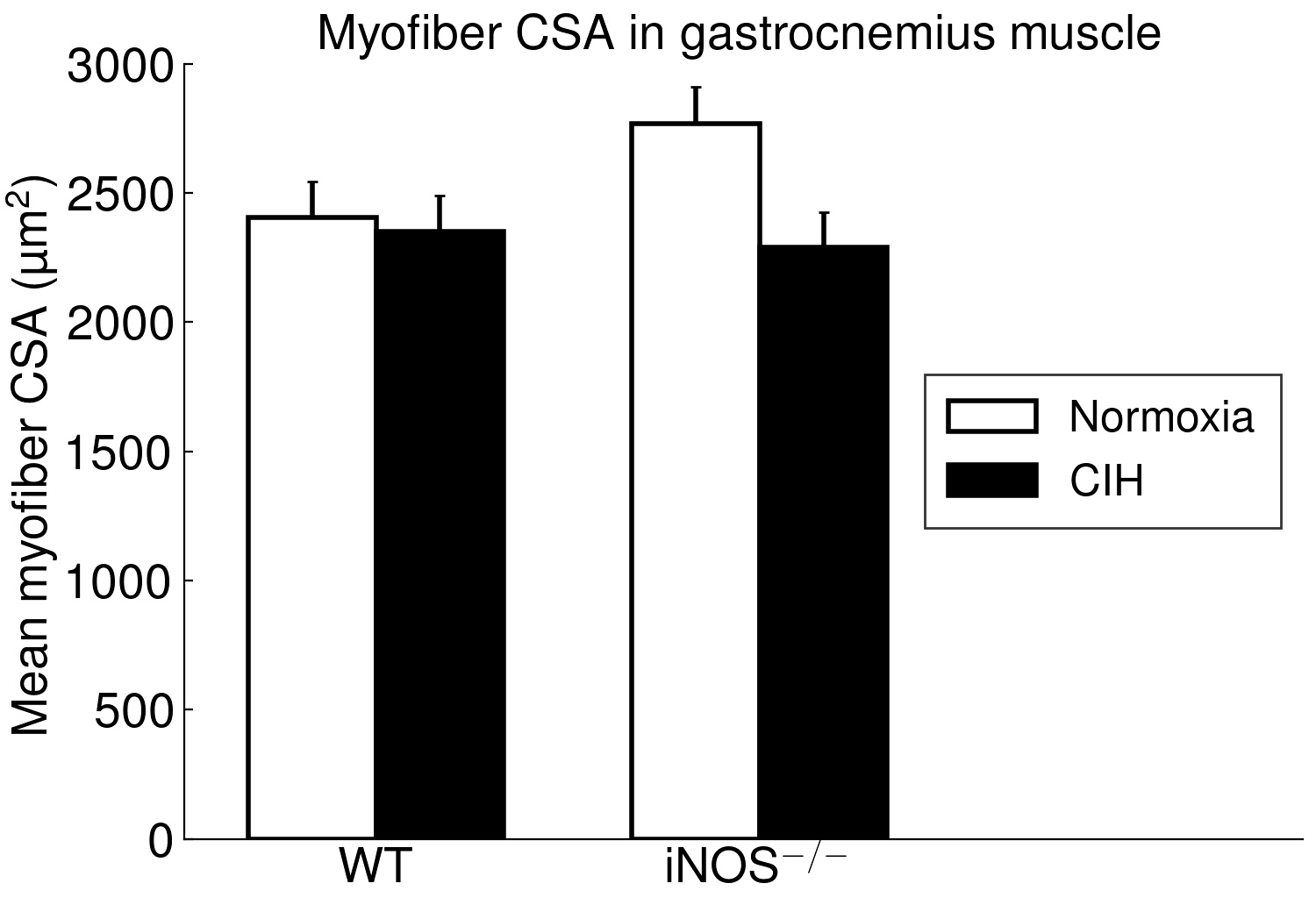

Supplement: Supplementary file 2 — Additional file 2. The myofiber CSA in gastrocnemius muscle is shown. Values are given as mean+SEM; n = 8 to 10 animals per group. [file 13395_2022_288_MOESM2_ESM.jpg]

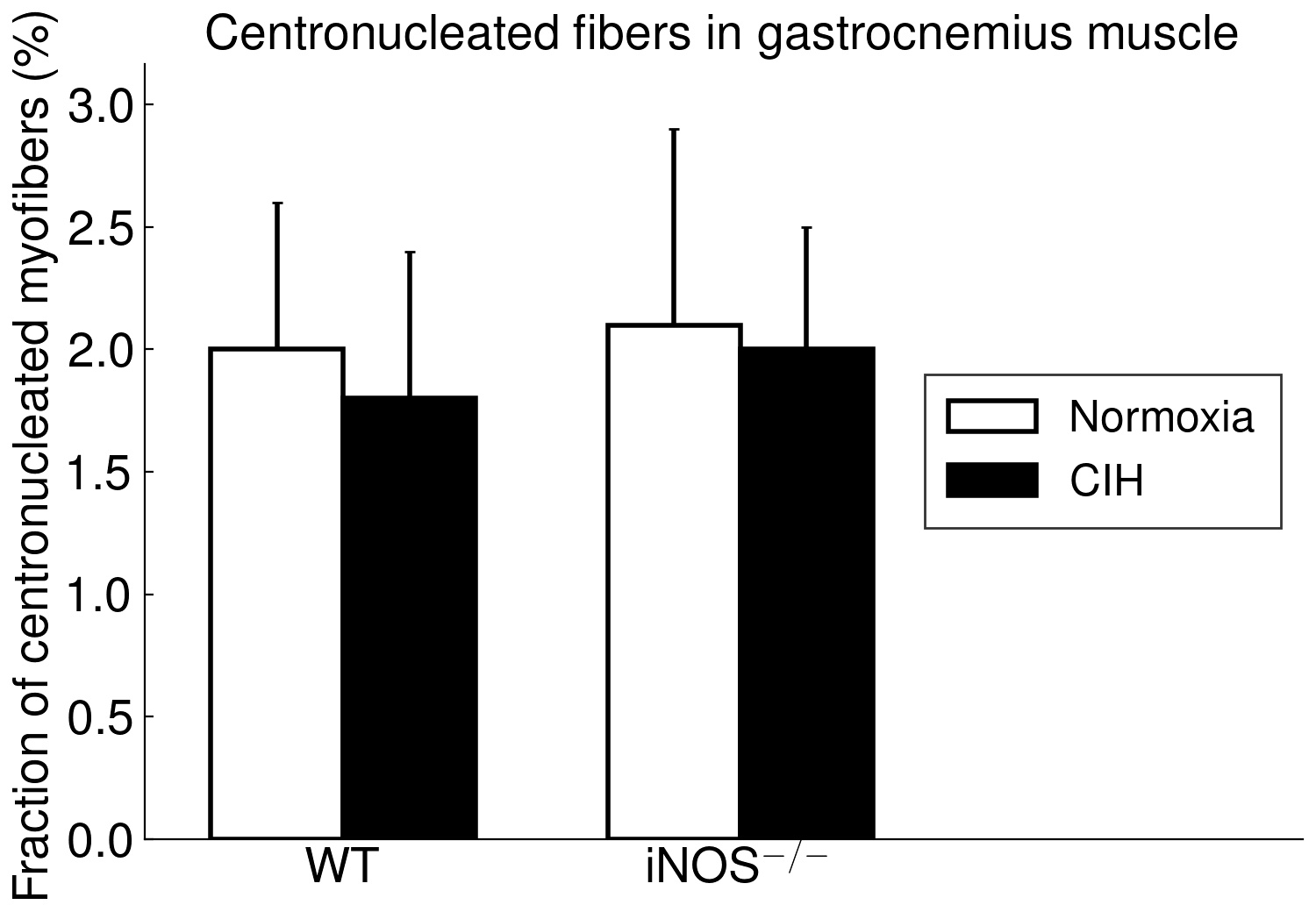

Supplement: Supplementary file 3 — Additional file 3. The percentage of centronucleated fibers in gastrocnemius muscle is shown. Values are given as mean+SEM; n = 8 to 10 animals per group. [file 13395_2022_288_MOESM3_ESM.jpg]

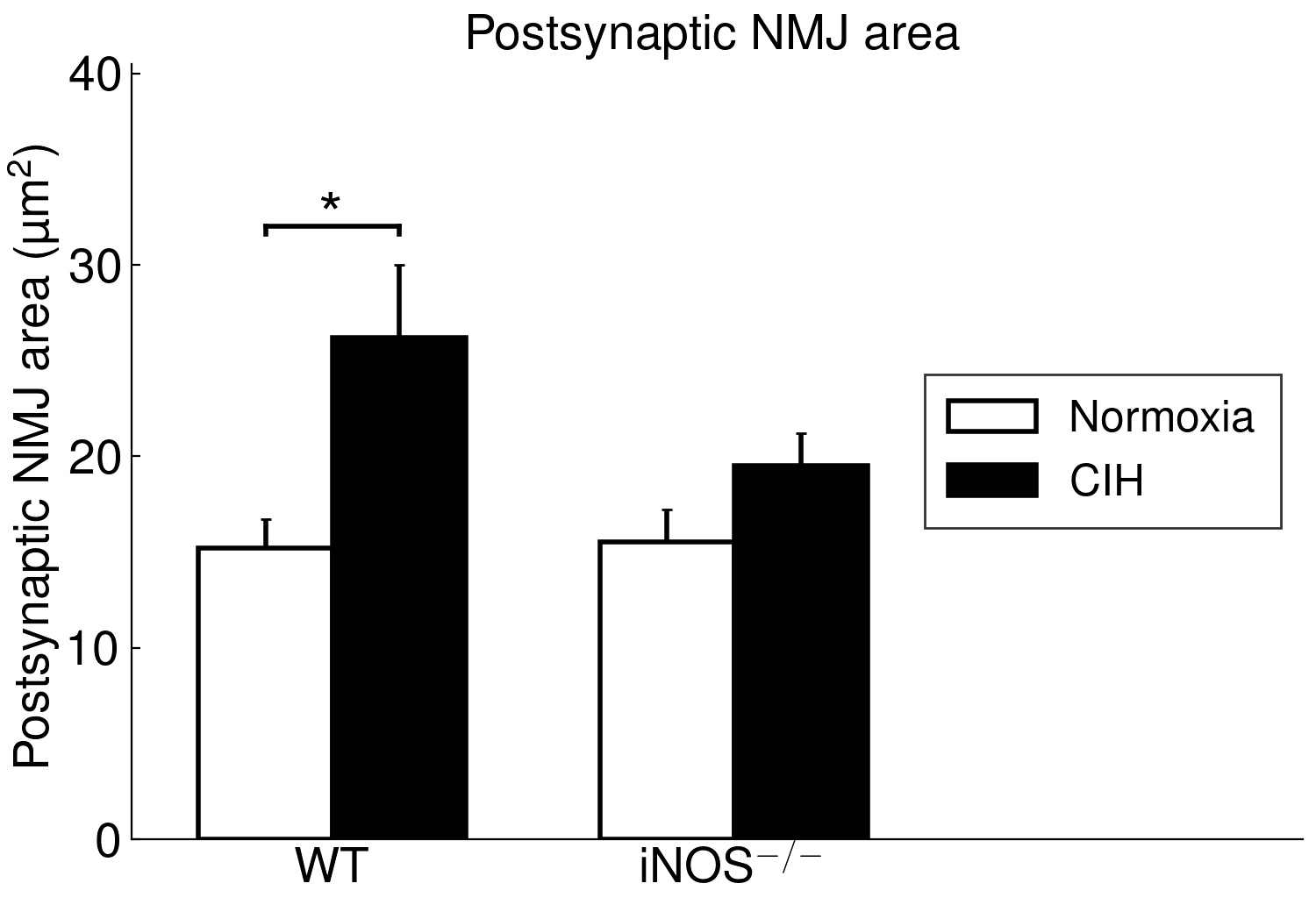

Supplement: Supplementary file 4 — Additional file 4. The post-synaptic NMJ area in gastrocnemius muscle is shown. Values are given as mean+SEM; n = 8 to 10 animals per group. * p<0.05, significance between CIH and NOX. [file 13395_2022_288_MOESM4_ESM.jpg]

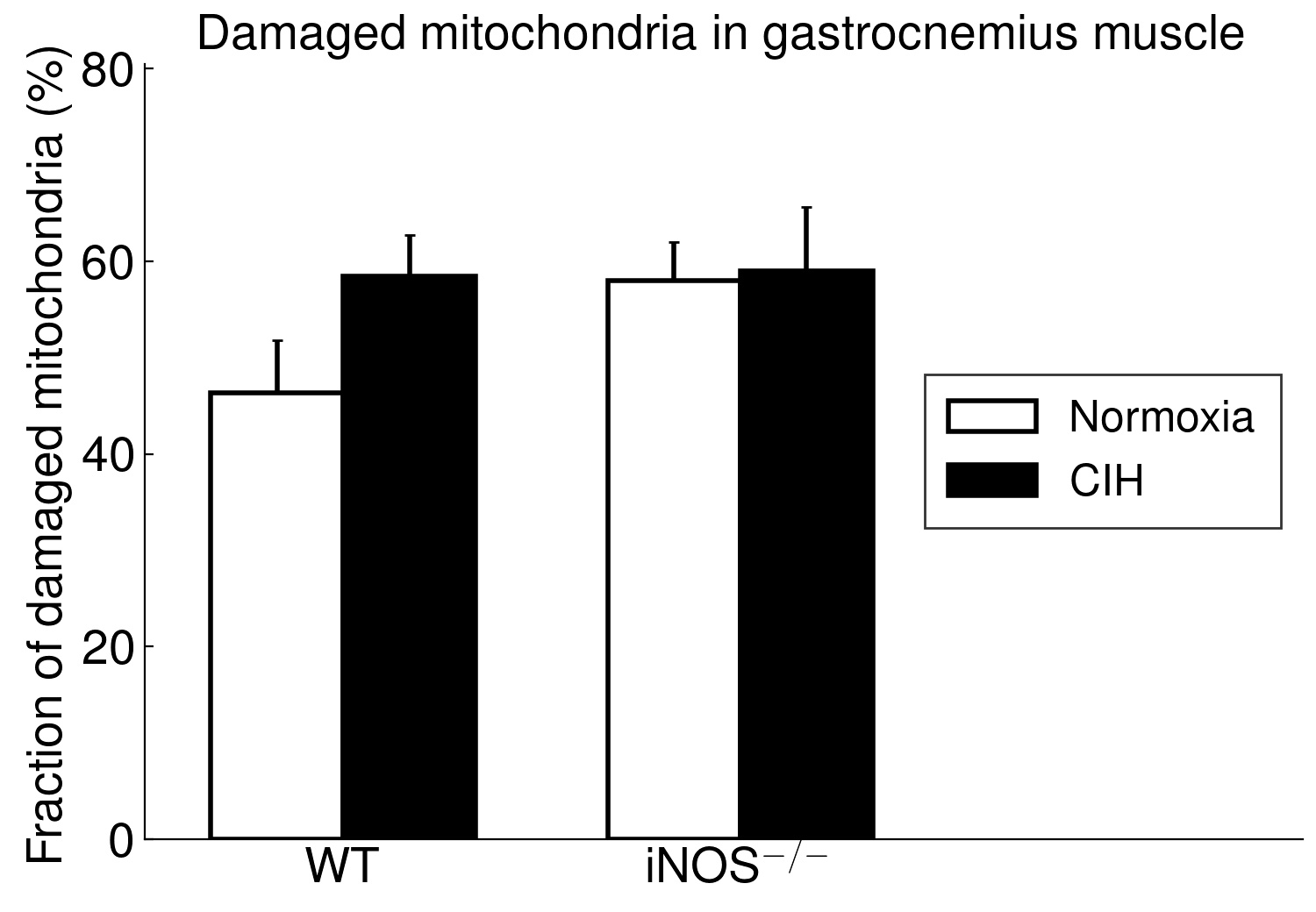

Supplement: Supplementary file 5 — Additional file 5. The percentage of damaged mitochondria in gastrocnemius muscle is shown. Values are given as mean+SEM; n = 8 to 10 animals per group. [file 13395_2022_288_MOESM5_ESM.jpg]

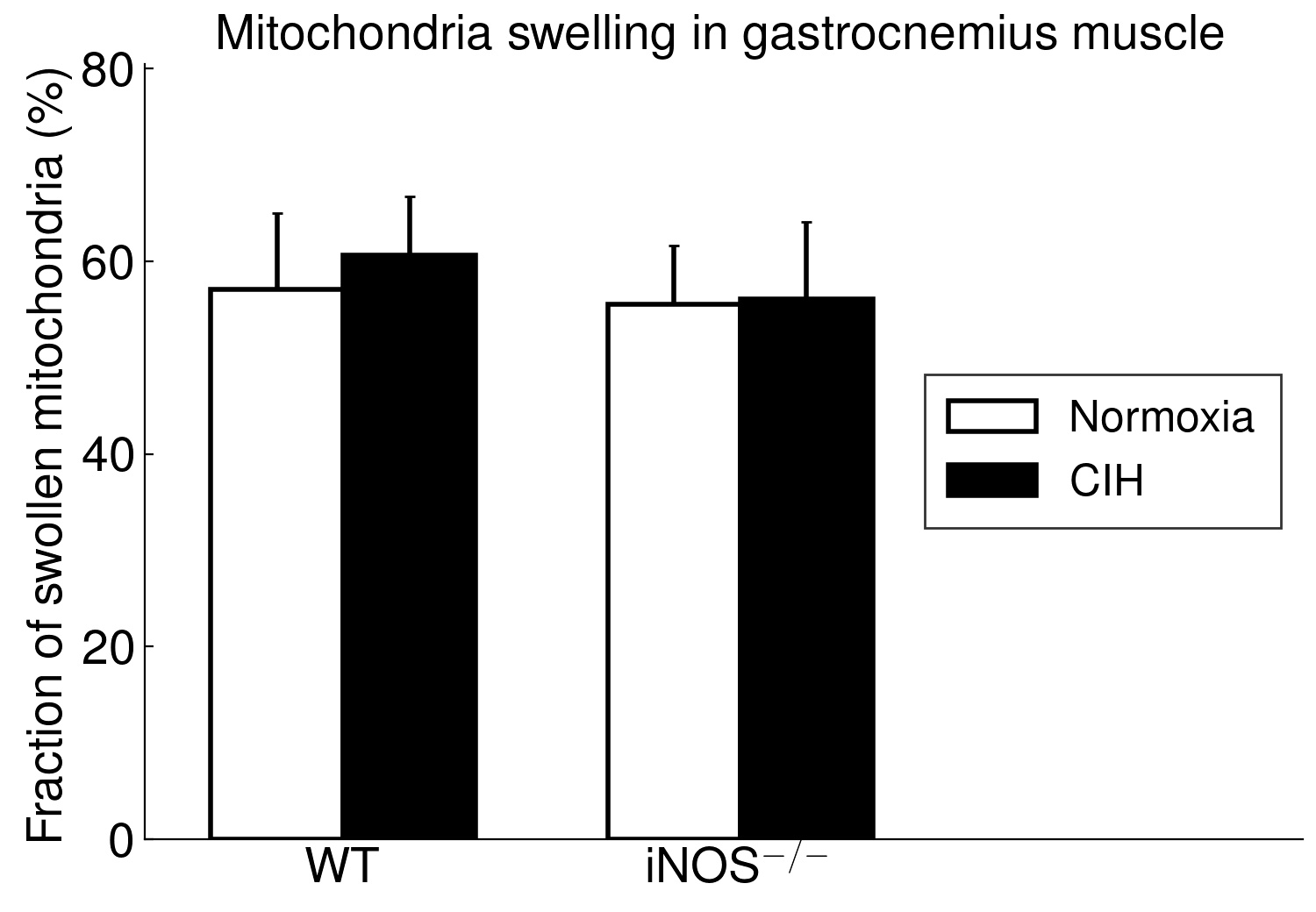

Supplement: Supplementary file 6 — Additional file 6. The percentage of swollen mitochondria in gastrocnemius muscle is shown. Values are given as mean+SEM; n = 8 to 10 animals per group. [file 13395_2022_288_MOESM6_ESM.jpg]
